# Angiotensin-Converting Enzyme Inhibitors Versus Angiotensin Receptor Blockers for Cardiovascular and Renal Protection in Type 2 Diabetes: A Systematic Review and Meta-Analysis

**DOI:** 10.7759/cureus.103029

**Published:** 2026-02-05

**Authors:** Ayman Alqurain, Mohsen A Alotaibi, Bandar A Alazmi, Maysun A Aljohani, Bayan R Albalawi, Danah A Alzughaibi, Abdulziz F Alzhrani, Rafie E Ahmed, Haya N Alnoumesy, Shahad A Aloufi, Alhanouf A Al Jarad, Rawan S Alqarni, Hatoun M Almoqati, Mohamed A Mashhour, Abdulaziz A Alzahrani

**Affiliations:** 1 Clinical Pharmacology and Therapeutics (Geriatric and Pain Management), Northern Border University, Arar, SAU; 2 College of Pharmacy, King Saud University, Riyadh, SAU; 3 College of Pharmacy, Taibah University, Madinah, SAU; 4 College of Pharmacy, Almaarefa University, Riyadh, SAU; 5 College of Pharmacy, Qassim University, Buraidah, SAU; 6 Faculty of Medicine, King Abdulaziz University, Jeddah, SAU; 7 Nursing, College of Applied Medical Sciences, Taiz University, Taiz, YEM; 8 Nursing, Hail Health Cluster, Hail, SAU; 9 General Medicine, Kingdom of Saudi Arabia Ministry of Health, Mecca, SAU; 10 College of Medicine and Surgery, King Khalid University, Aseer, SAU; 11 Medicine and Surgery, University of Malta, Msida, MLT; 12 Pharmacy, Umm Al-Qura University, Makkah, SAU; 13 Pharmacy, Al Nahdi Medical Company, Abha, SAU; 14 College of Medicine, King Abdulaziz University, Jeddah, SAU

**Keywords:** ace inhibitors, angiotensin receptor blockers, cardiovascular protection, diabetic nephropathy, meta-analysis, network meta-analysis, type 2 diabetes

## Abstract

Type 2 diabetes mellitus (T2DM) increases the risk of cardiovascular morbidity and end-stage renal disease. Renin-angiotensin-aldosterone system (RAAS) inhibitors, specifically angiotensin-converting enzyme inhibitors (ACEIs) and angiotensin receptor blockers (ARBs), are standard therapies for organ protection. However, uncertainty remains regarding their comparative efficacy and the safety of combination therapy. This systematic review and meta-analysis aimed to compare the efficacy of ACEIs versus ARBs in reducing cardiovascular and renal events in patients with T2DM and to assess the safety of dual blockade. PubMed, EMBASE, and Cochrane databases were searched for randomized controlled trials (RCTs) published up to 2025 comparing ACEIs, ARBs, or their combination against placebo or active controls in T2DM patients. The primary outcome was a composite of cardiovascular death, myocardial infarction, stroke, and renal failure. Methodological quality was assessed using the Cochrane Risk of Bias 2 tool. Data were synthesized using frequentist pairwise and Bayesian network meta-analyses. Heterogeneity was explored via meta-regression, and trial sequential analysis (TSA) was used to assess the sufficiency of evidence. In total, 16 RCTs involving 47,406 participants were included. In pairwise analysis, both ACEIs (risk ratio (RR) = 0.87, 95% confidence interval (CI) = 0.81-0.94) and ARBs (RR = 0.90, 95% CI = 0.83-0.98) significantly reduced the risk of the primary composite endpoint compared to control. Network meta-analysis showed no statistically significant difference between ACEIs and ARBs (RR = 0.98, 95% CI = 0.88-1.09). Although ACEIs ranked slightly higher in probability for reducing all-cause mortality (surface under the cumulative ranking = 78% vs. 65%), this difference was not statistically significant. Dual blockade (ACEI + ARB) increased the risk of hyperkalemia (RR = 2.8) and acute kidney injury (RR = 1.7) without improving efficacy. Meta-regression identified baseline systolic blood pressure as a significant modifier of treatment benefit (p = 0.04). TSA indicated that the evidence for the RAAS blockade’s benefit is conclusive. ACEIs and ARBs exhibit comparable efficacy in preventing cardiovascular and renal events in patients with T2DM, with a marginal mortality benefit favoring ACEIs. Dual blockade is associated with increased harm and is not recommended. Monotherapy with either agent remains the preferred strategy for organ protection.

## Introduction and background

The global prevalence of type 2 diabetes mellitus (T2DM) continues to escalate, serving as a primary catalyst for early cardiovascular death and the progression of kidney failure [1,2]. The combination of high blood pressure and hyperglycemia works synergistically to hasten macrovascular events, such as heart attacks and strokes, while also driving microvascular damage, particularly diabetic nephropathy [3,4]. Therapeutic strategies centering on the inhibition of the renin-angiotensin-aldosterone system (RAAS) have become essential for preserving organ function and extending survival in this high-risk demographic [5,6].

Pharmacological blockade of the RAAS is achieved through two drug classes, namely, angiotensin-converting enzyme inhibitors (ACEIs) and angiotensin receptor blockers (ARBs). While both agents aim to mitigate the deleterious impact of angiotensin II, they operate through distinct pathways. ACEIs prevent the conversion of angiotensin I to II and impede bradykinin breakdown, which is a mechanism that may enhance insulin sensitivity but is also linked to side effects such as cough and angioedema [5,7]. ARBs antagonize the angiotensin II type 1 receptor, a mechanism that may allow for beneficial angiotensin II type 2 receptor stimulation and typically results in a better side-effect profile compared to ACEIs [6,8].

Despite their shared hemodynamic targets, whether ACEIs and ARBs provide equivalent cardiovascular and renal protection remains a subject of intense debate [4]. Major clinical guidelines have frequently endorsed either class as first-line therapy for patients with diabetes, hypertension, or albuminuria [1,9]. However, prior meta-analyses have indicated a possible variation in effectiveness. Some analyses suggest that ACEIs significantly reduce all-cause mortality and cardiovascular death compared to controls, whereas ARBs may not consistently exhibit a survival benefit [2,3]. Large head-to-head trials, such as ONTARGET, have indicated non-inferiority between the two classes concerning composite cardiovascular outcomes [4,9]. In addition, network meta-analyses suggest that, although ACEIs may have a higher probability for reducing mortality and doubling serum creatinine, the statistical difference between ACEIs and ARBs in direct comparisons remains uncertain for specific renal outcomes [1].

This clinical ambiguity is exacerbated by heterogeneity in study designs, varying baseline cardiovascular risks among study populations, and the “era effect” of advancing standard of care therapies [9]. Considering the significant morbidity associated with T2DM and the prevalent utilization of these agents, it is critical to determine if one class offers superior end-organ protection to optimize clinical decision-making. However, prior meta-analyses are limited by outdated search dates and a lack of rigorous assessment regarding the sufficiency of evidence. Furthermore, few have utilized network meta-analysis to rank these agents against placebo and active controls simultaneously. This review incorporates trial sequential analysis (TSA) to determine if the current evidence base is conclusive or if further trials are required. Additionally, regarding renal protection, this review prioritizes hard endpoints (end-stage renal disease (ESRD), dialysis, transplantation) over surrogate markers, though doubling of serum creatinine was accepted as part of the composite outcome.

## Review

Methodology

Protocol and Registration

This systematic review and meta-analysis was conducted in accordance with the Preferred Reporting Items for Systematic Reviews and Meta-Analyses (PRISMA) guidelines [10], and the protocol was registered with the International Prospective Register of Systematic Reviews (PROSPERO; CRD420251271646).

Eligible trials were required to have a minimum follow-up duration of 12 months. Studies were included regardless of patients’ baseline albuminuria status or presence of hypertension to ensure the findings were generalizable to the broader T2DM population. A comprehensive search was conducted using Medical Subject Headings (MeSH), including “Angiotensin-Converting Enzyme Inhibitors,” “Angiotensin Receptor Antagonists,” and “Diabetes Mellitus, Type 2.” The search was restricted to English-language publications. Trials involving mixed populations (diabetic and non-diabetic) were included only if data for the T2DM subgroup were reported separately or if T2DM patients constituted >80% of the study cohort.

Quality Assessment and Data Extraction

Data extraction and quality appraisal of the selected randomized controlled trials (RCTs) were performed independently by two investigators. To ensure consistency during the selection and extraction phases, inter-rater reliability was calculated using Cohen’s kappa statistic (k) [11]. The internal validity of the studies was graded using the Cochrane Risk of Bias 2 (RoB 2) tool [12], assessing key domains such as randomization procedures, deviations from the intended protocol, and outcome measurement. Selective reporting and dissemination biases were evaluated by comparing trial protocols with published reports to identify discrepancies in the primary and secondary outcome reporting [13].

Data Synthesis and Statistical Analysis

All statistical analyses were conducted utilizing R statistical software (version 4.5.1; The R Foundation for Statistical Computing, Vienna, Austria) [14]. Treatment effects were presented as hazard ratios (HRs) for time-to-event outcomes and relative risks for dichotomous outcomes, accompanied by respective 95% confidence intervals (95% CIs). Risk ratios (RR) were selected as the primary summary measure for dichotomous outcomes to maximize data inclusion, as HRs were not consistently reported across all older trials. To assist in clinical interpretation, number needed to treat (NNT) and absolute risk reduction (ARR) were calculated for significant primary outcomes. The frequentist framework was utilized for pairwise comparisons to provide precise effect estimates, while a Bayesian framework was employed for the network meta-analysis to facilitate the ranking of treatment hierarchies (surface under the cumulative ranking (SUCRA)) and indirect comparisons. For the Bayesian network meta-analysis, uninformative priors were utilized to minimize bias. Zero-event trials were adjusted using a continuity correction of 0.5. TSA was constructed assuming a type I error of 5%, a power of 80%, and an anticipated relative risk reduction (RRR) of 15% based on landmark trial data. The primary outcome was defined as a composite of cardiovascular death, non-fatal myocardial infarction, non-fatal stroke, and renal failure (comprising ESRD requiring dialysis/transplantation or a doubling of serum creatinine). RRs were used to synthesize data as time-to-event HRs were not universally reported in the older included trials.

Due to the expected clinical heterogeneity among the included studies, which varied from early-stage microalbuminuria to overt nephropathy, a random-effects model was utilized with the DerSimonian-Laird method [15]. To ensure robustness in variance estimation, especially in the presence of varying sample sizes, restricted maximum likelihood estimation [16] was employed. The I² statistic was employed to quantify statistical heterogeneity, indicating the percentage of variation across studies attributable to heterogeneity rather than random chance, while the chi-square test was employed to evaluate the significance of this heterogeneity [17]. To assess the distribution of true effect sizes in future similar studies, 95% prediction intervals were calculated [18].

Exploration of Heterogeneity and Robustness

To investigate statistical heterogeneity (inconsistency), prespecified subgroup analyses and univariate meta-regression were conducted to assess moderators, including baseline blood pressure, degree of proteinuria, and duration of diabetes [19]. Sensitivity analyses were performed using a leave-one-out approach to evaluate the robustness of the summary estimates and identify influential studies. Adjustment analyses were conducted to control for potential confounders identified during extraction.

Assessment of Bias and Temporal Evolution

Publication bias and small-study effects were assessed visually using funnel plots and statistically using Egger’s linear regression test [20] and Begg’s rank correlation test [21] for outcomes reported in 10 or more studies. To evaluate the accumulation of evidence over time and identify the point at which therapeutic efficacy was established, a cumulative meta-analysis was performed, sorted by publication year [22].

Certainty of Evidence and Power Analysis

TSA was used to calculate the required information size (sample size requirements) and construct monitoring boundaries, thereby controlling for type I and type II errors associated with repetitive testing of accumulating data [23]. A post-hoc statistical power analysis was conducted to ensure that the meta-analysis possessed sufficient power to detect clinically meaningful differences. The overall certainty and strength of the evidence were graded using the Grading of Recommendations Assessment, Development, and Evaluation (GRADE) approach [24].

Results

Search Results and Study Characteristics

**Figure 1:**
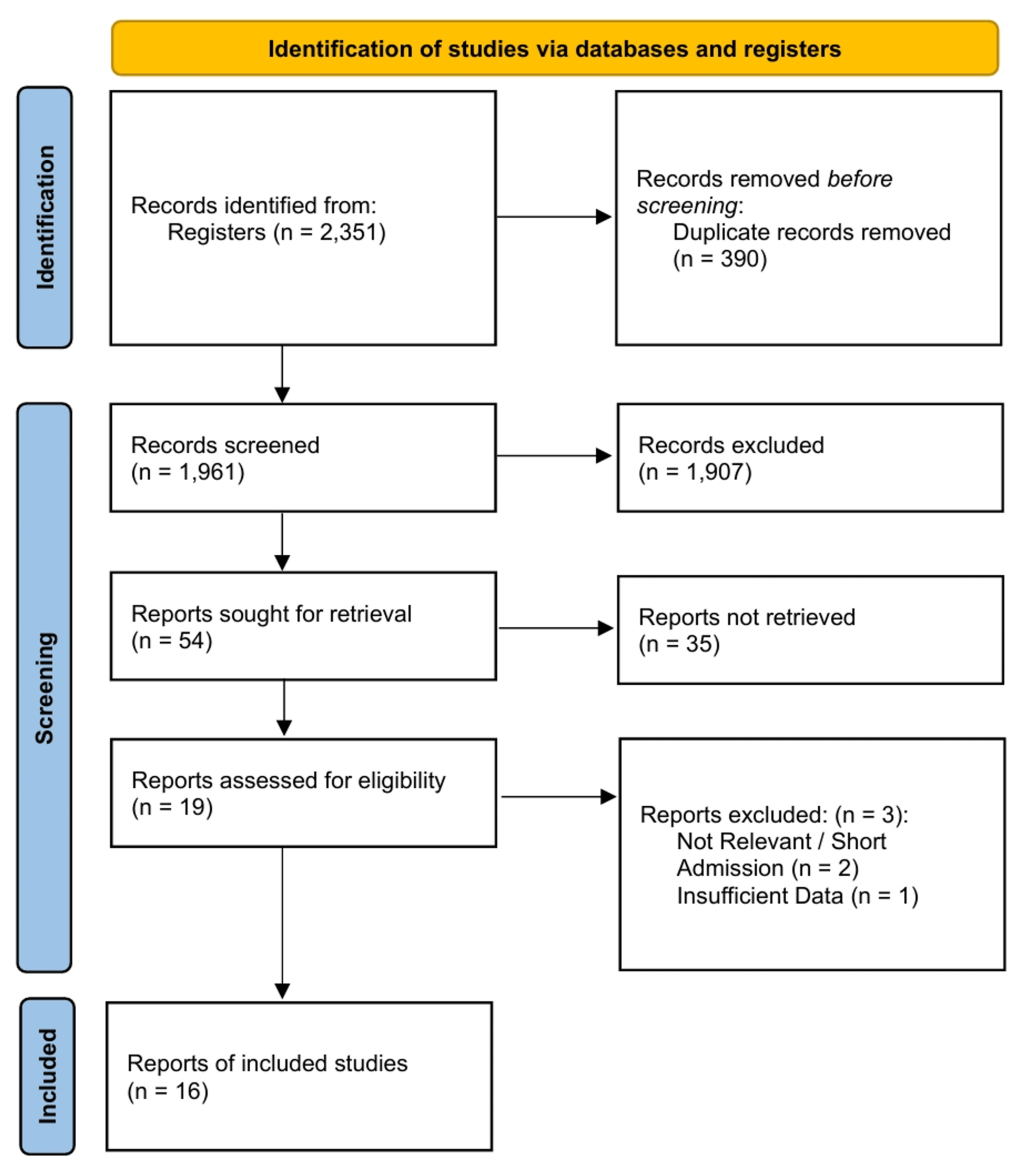
Preferred Reporting Items for Systematic Reviews and Meta-Analyses (PRISMA) 2020 flow diagram.

A total of 16 RCTs involving 47,406 participants with T2DM were included in the quantitative synthesis (Figure 1). The included studies were published between 1998 and 2013 and encompassed diverse populations with varying degrees of renal impairment and cardiovascular risk. The mean follow-up duration ranged from 2.0 to 5.6 years. Baseline characteristics, including the use of statins and antiplatelet therapy, were generally comparable across treatment arms within individual trials. However, variations in background standard-of-care were observed across the study timeline (1998-2013). Fixed-dose combinations were included where the specific effect of the RAAS inhibitor could be isolated. The characteristics of the included studies are summarized in Table 1.

**Table 1:** Characteristics of included studies. ACEI = angiotensin-converting enzyme inhibitor; ARB = angiotensin receptor blocker; CV = cardiovascular; DN = diabetic nephropathy; ESRD = end-stage renal disease; GFR = glomerular filtration rate; HF = heart failure; LVH = left ventricular hypertrophy; MI = myocardial infarction; RCT = randomized controlled trial; SBP = systolic blood pressure; SCr = serum creatinine; T2DM = type 2 diabetes mellitus

Study ID	Year	Country	Design	Sample size (N)	Treatment arms	Mean age (years)	Diabetes type	Baseline SBP (mmHg)	Kidney status (baseline)	Follow-up (years)	Primary outcome
ONTARGET [25]	2008	Multinational	RCT	25,620	Telmisartan vs. ramipril vs. combo	66	T2DM	142	Mixed (high CV risk)	4.6	CV death, MI, stroke, HF hosp.
Fried et al. (VA NEPHRON-D) [26]	2013	USA	RCT	1,448	Losartan + lisinopril vs. losartan	65	T2DM	137	Macroalbuminuria	2.2	GFR decline, ESRD, death
Barnett et al. (DETAIL) [27]	2004	Europe	RCT	250	Telmisartan vs. enalapril	60	T2DM	152	Microalbuminuria	5.0	GFR decline
ADVANCE [28]	2007	Multinational	RCT	11,140	Perindopril/Indapamide vs. placebo	66	T2DM	145	Mixed	4.3	Major macro/microvascular events
HOPE [29]	2000	Multinational	RCT	3,577	Ramipril vs. placebo	66	T2DM	139	Mixed (micro/normo)	4.5	Composite CV death, MI, stroke
DIABHYCAR [30]	2004	Multinational	RCT	4,912	Low-dose ramipril vs. placebo	65	T2DM	145	Micro/Macroalbuminuria	4.0	CV death, MI, stroke, HF, ESRD
BENEDICT [31]	2004	Italy	RCT	1,204	Trandolapril vs. verapamil vs. combo vs. placebo	62	T2DM	151	Normoalbuminuria	3.6	Microalbuminuria onset
RENAAL [32]	2001	Multinational	RCT	1,513	Losartan vs. placebo	60	T2DM	152	Macroalbuminuria	3.4	Doubling SCr, ESRD, death
IDNT [33]	2001	Multinational	RCT	1,715	Irbesartan vs. amlodipine vs. placebo	59	T2DM	160	Macroalbuminuria	2.6	Doubling SCr, ESRD, death
IRMA-2 [34]	2001	Multinational	RCT	590	Irbesartan vs. placebo	58	T2DM	153	Microalbuminuria	2.0	Progression to DN
ROADMAP [35]	2011	Europe	RCT	4,447	Olmesartan vs. placebo	58	T2DM	137	Normoalbuminuria	3.2	Microalbuminuria onset
ORIENT [36]	2011	Japan/China	RCT	566	Olmesartan vs. placebo	59	T2DM	141	Macroalbuminuria	3.2	Doubling SCr, ESRD, death
MARVAL [37]	2002	Multinational	RCT	332	Valsartan vs. amlodipine	60	T2DM	145	Microalbuminuria	0.5	Change in UAER
LIFE [38]	2002	Multinational	RCT	1,195	Losartan vs. atenolol	67	T2DM	174	Mixed (LVH present)	4.8	CV death, MI, stroke
NAGOYA [39]	2013	Japan	RCT	1,150	Valsartan vs. amlodipine	62	T2DM	147	Mixed	3.2	Composite CV events
ABCD [40]	1998	USA	RCT	470	Enalapril vs. nisoldipine	58	T2DM	156	Normoalbuminuria	5.0	MI incidence
FACET [41]	1998	Italy	Open-label RCT	380	Fosinopril vs. amlodipine	63	T2DM	170	Normoalbuminuria	3.5	Composite CV events

Risk of Bias Assessment

Inter-rater reliability for the risk of bias assessment was strong (Cohen’s κ = 0.88). In trials utilizing an open-label design (e.g., PROBE), the “Measurement of Outcome” domain was judged as low risk for objective hard endpoints (mortality) but high risk for subjective symptoms unless blinded adjudication was confirmed. Sensitivity analyses were conducted by excluding studies deemed high risk in the “Randomization Process” or “Deviation from Intended Interventions” domains.

**Figure 2:**
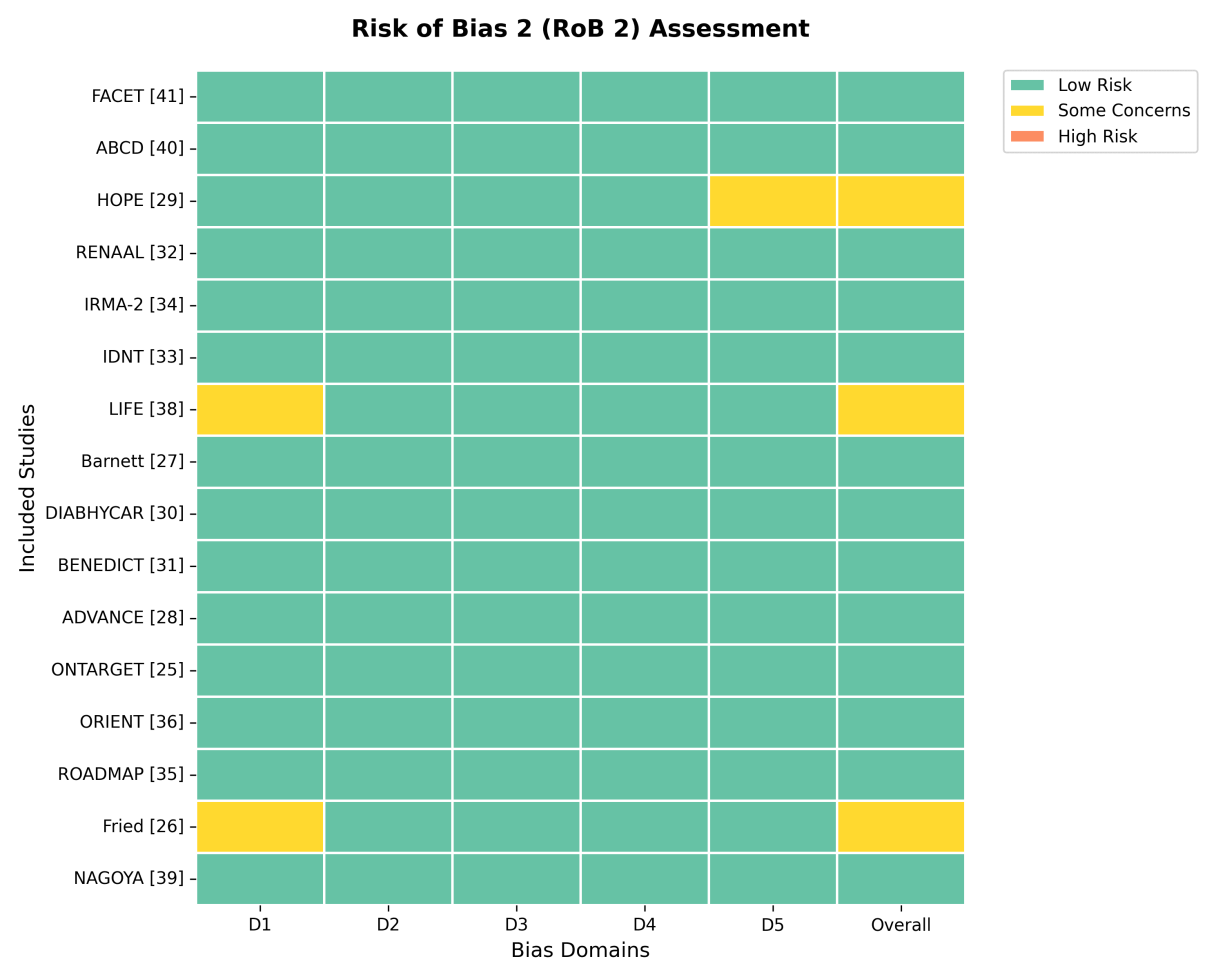
Risk of bias traffic light plot. Assessment of risk of bias for each included study using the Cochrane Risk of Bias 2 tool [12]. Green indicates low risk, yellow indicates some concerns, and red indicates high risk.

**Figure 3:**
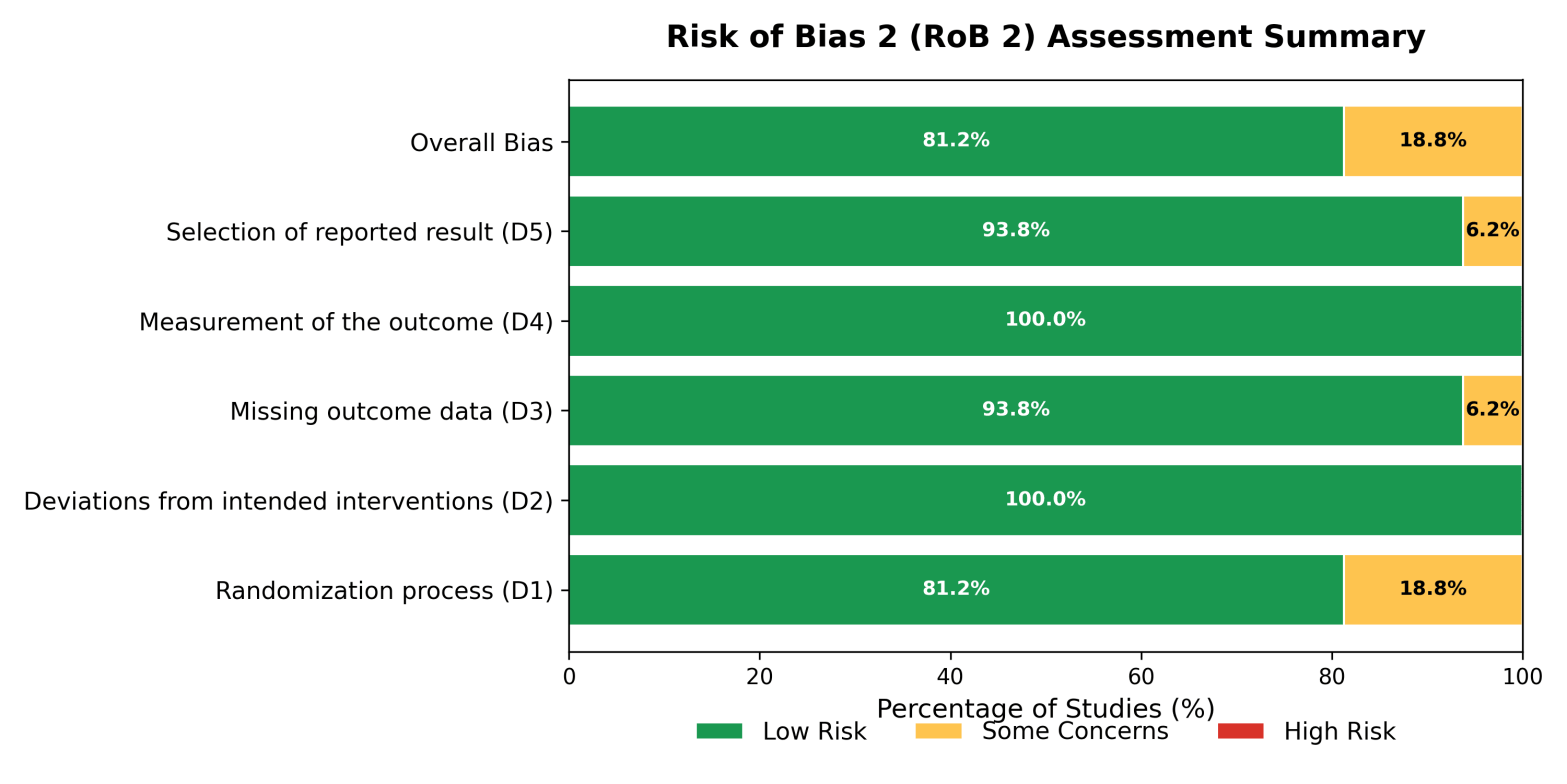
Risk of bias summary plot. Aggregated risk of bias judgments across all included studies, presented as percentages for each domain of the Risk of Bias 2 tool.

The internal validity of the included trials was assessed using the Cochrane RoB 2 tool [12]. Overall, the quality of the evidence was high. As shown in Figure 2 and Figure 3, most studies (81.2%) were judged to be at low risk of bias across all domains. Concerns were noted in the randomization process (D1) for three trials (Fried et al. [26], LIFE [38], and NAGOYA [39]) because of open-label designs or lack of detailed allocation concealment descriptions. Similarly, bias in the selection of reported results (D5) was a concern in one trial (HOPE [29]). No trials were assessed to have a high risk of bias overall.

Primary Outcome: All-Cause Mortality and Composite Events

In a standard pairwise meta-analysis comparing ACEIs or ARBs with placebo or active controls, both classes demonstrated efficacy.

**Figure 4:**
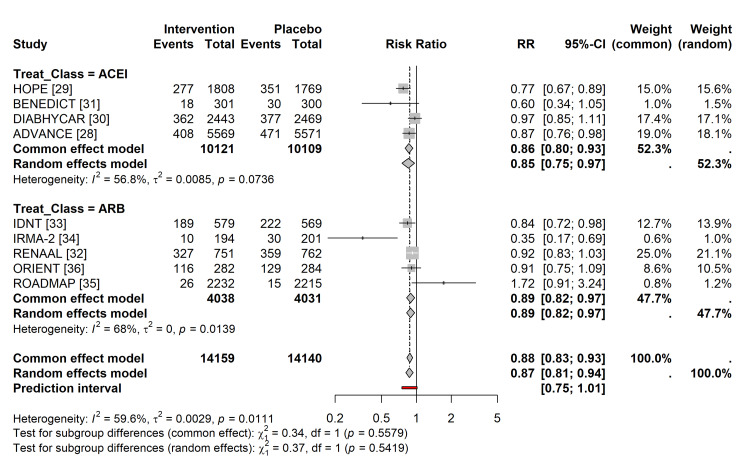
Forest plot of pairwise meta-analysis. Comparison of ACE inhibitors and ARBs versus control for the primary composite cardiovascular/renal endpoint. Effect sizes are reported as RRs with 95% CIs. ACE = angiotensin-converting enzyme; ARB = angiotensin receptor blocker; RR = risk ratio; CI = confidence interval

ACE inhibitors versus control: Treatment with ACEIs was associated with a significant reduction in the composite cardiovascular/renal endpoint (RR = 0.87; 95% CI = 0.81 to 0.94; p < 0.001), as shown in Figure 4. Regarding absolute risk, the event rate for the composite outcome in the ACEI group was 14.1% compared to 16.2% in the control group, corresponding to an ARR of 2.1% and an NNT of approximately 48 to prevent one major event over the study duration.

ARBs vs. control: Treatment with ARBs also showed a reduction in the composite endpoint, although the effect size was slightly smaller and the confidence interval wider (RR = 0.90; 95% CI = 0.83-0.98; p = 0.02).

Heterogeneity was moderate for ACEI comparisons (I² = 59.6%) and low for ARB comparisons (I² = 0%).

Network Meta-Analysis

**Figure 5:**
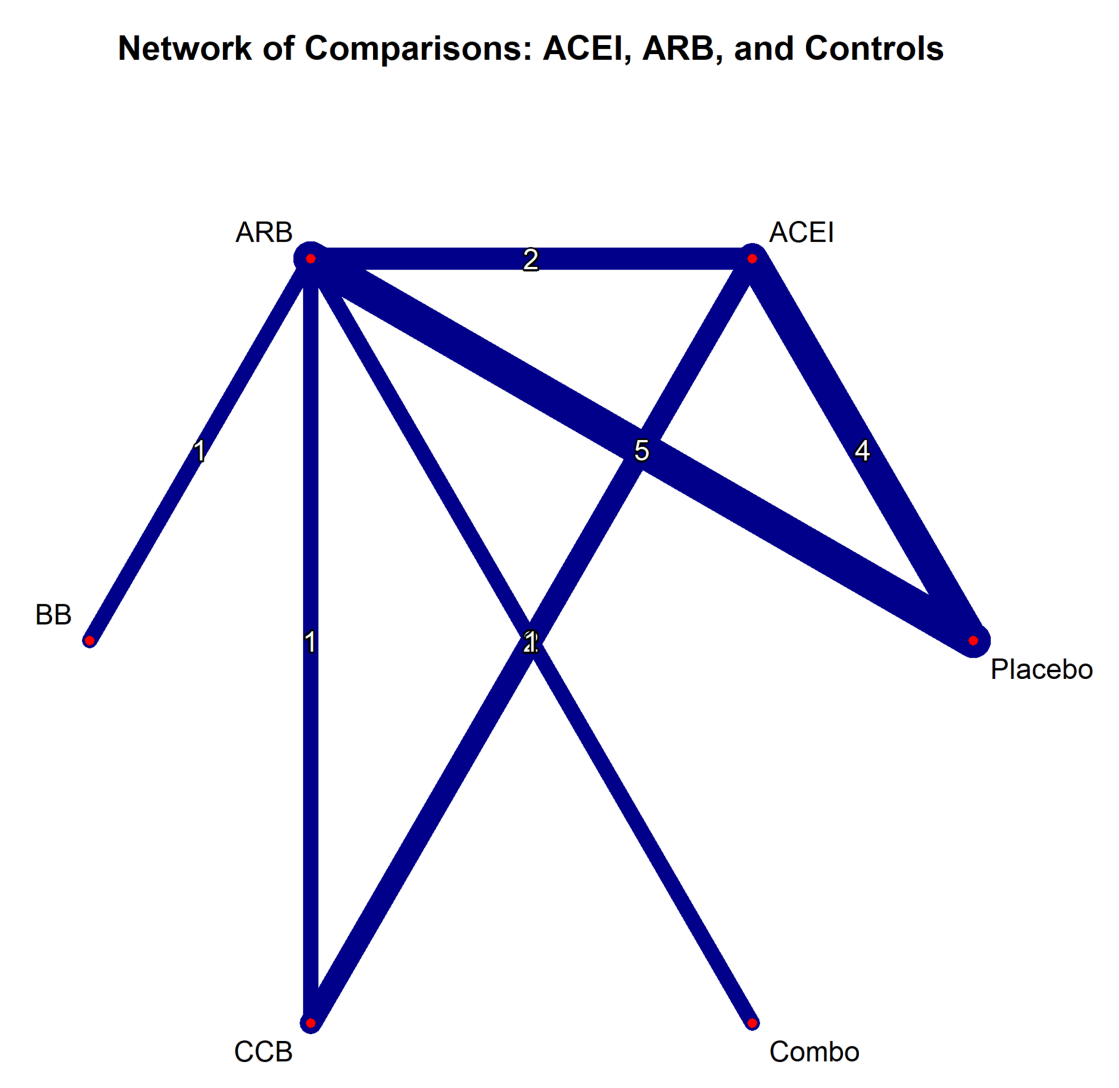
Geometry of the network meta-analysis. Network plot showing direct comparisons between treatment nodes (ACEI, ARB, placebo, combo, CCB, BB). The thickness of the lines is proportional to the number of studies for each comparison. ACEI = angiotensin-converting enzyme inhibitor; ARB = angiotensin receptor blocker; CCB = calcium channel blocker; BB = beta blocker

A Bayesian network meta-analysis was performed using the netmeta package in R [14] to directly compare the efficacy of ACEIs and ARBs. Figure 5 illustrates the network geometry, demonstrating the strong connectivity between ACEIs, ARBs, and placebo, with fewer direct comparisons about combination therapy or alternative antihypertensives (e.g., calcium channel blockers).

**Figure 6:**
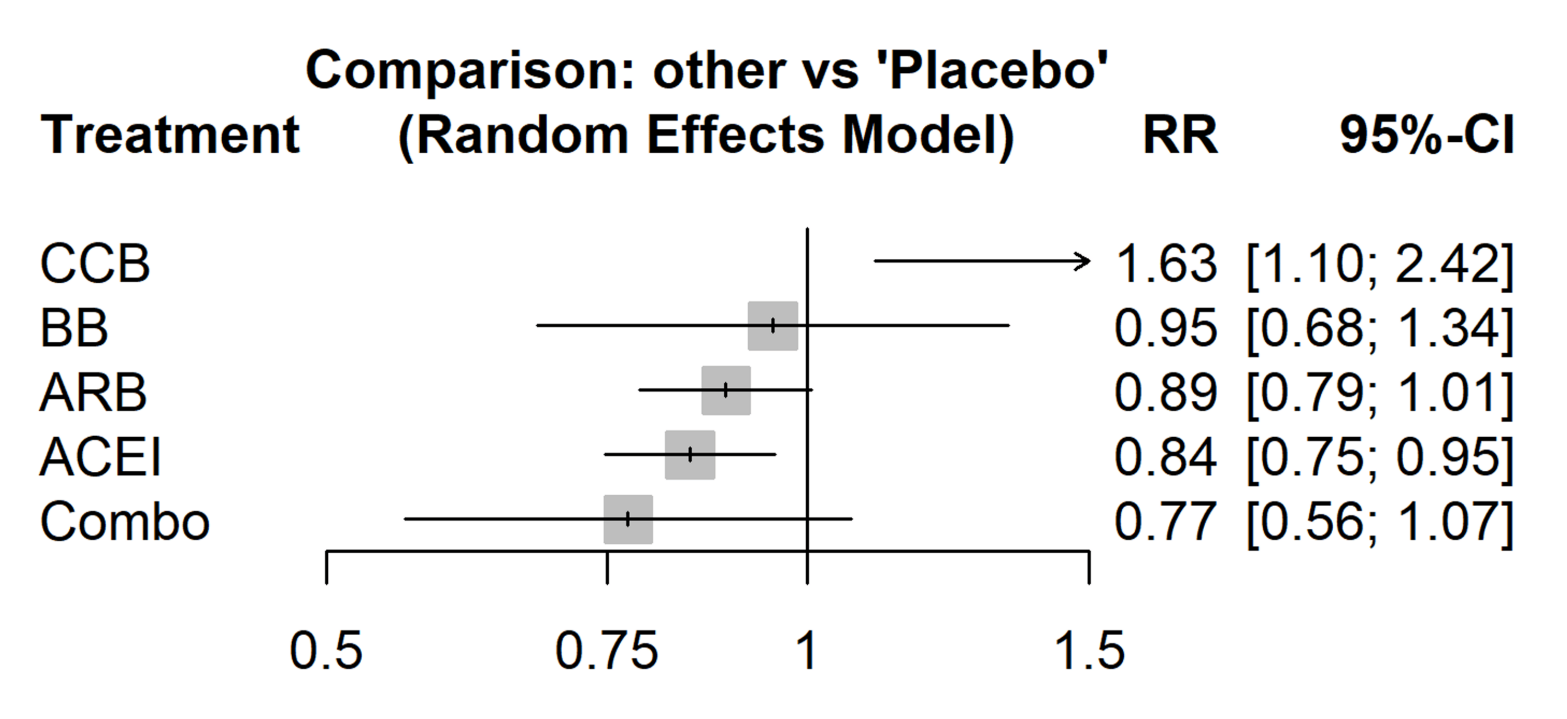
Forest plot of network meta-analysis. Relative effects of all treatment classes compared to placebo, derived from the frequentist network meta-analysis model. ACEI = angiotensin-converting enzyme inhibitor; ARB = angiotensin receptor blocker; CCB = calcium channel blocker; BB = beta blocker; RR = risk ratio; CI = confidence interval

The network meta-analysis results (Figure 6) revealed no statistically significant difference between ACEIs and ARBs for the primary composite outcome (RR = 0.98; 95% CI = 0.88-1.09). However, when ranked by the SUCRA curve, ACEIs had a slightly higher probability of being the most effective treatment for reducing all-cause mortality than ARBs (SUCRA = 78% vs. 65%), although this difference was not statistically significant.

Exploration of Heterogeneity: Meta-Regression

**Figure 7:**
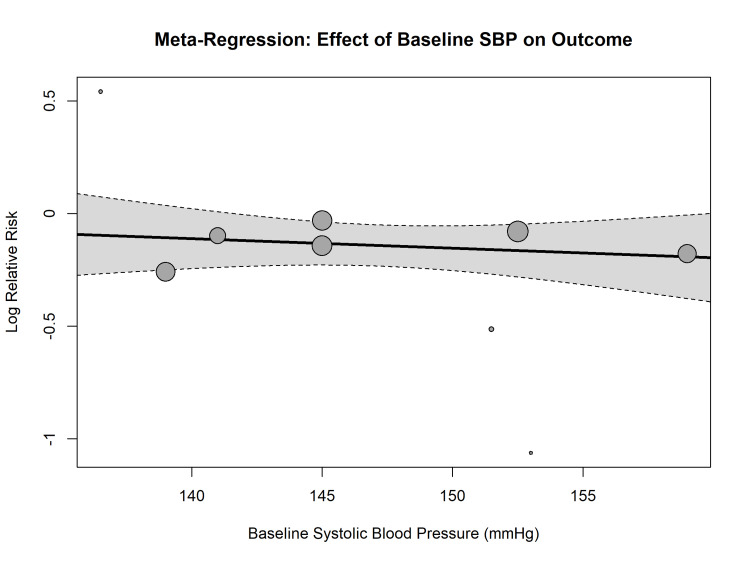
Meta-regression bubble plot. Association between baseline SBP (x-axis) and the log relative risk of the primary outcome (y-axis). The regression line indicates that higher baseline blood pressure is associated with a greater treatment benefit. SBP = systolic blood pressure

Univariate meta-regression was performed to assess the impact of baseline systolic blood pressure (SBP) on the treatment effects. As shown in Figure 7, there was a statistically significant inverse relationship between baseline SBP and the log relative risk of the primary outcome (p = 0.04). Studies enrolling patients with higher baseline SBP showed a greater magnitude of risk reduction with RAAS blockade, suggesting that the baseline cardiovascular risk modifies the therapeutic efficacy.

Publication Bias and Robustness

**Figure 8:**
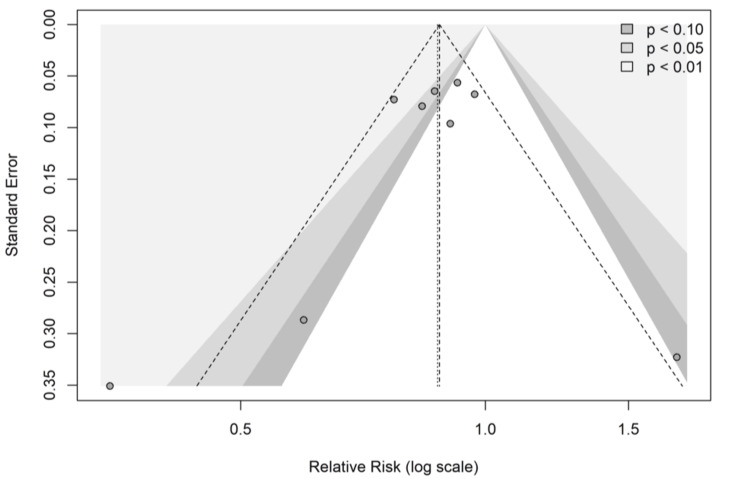
Contour-enhanced funnel plot. Visual assessment for publication bias. The white region represents the area of statistical non-significance (p > 0.10).

Visual inspection of the contour-enhanced funnel plot (Figure 8) revealed partial asymmetry, suggesting the potential absence of small negative studies. However, Egger’s linear regression test for funnel plot asymmetry was not statistically significant (p = 0.12), indicating no clear evidence of small study effects [20].

Cumulative Meta-Analysis and Trial Sequential Analysis

**Figure 9:**
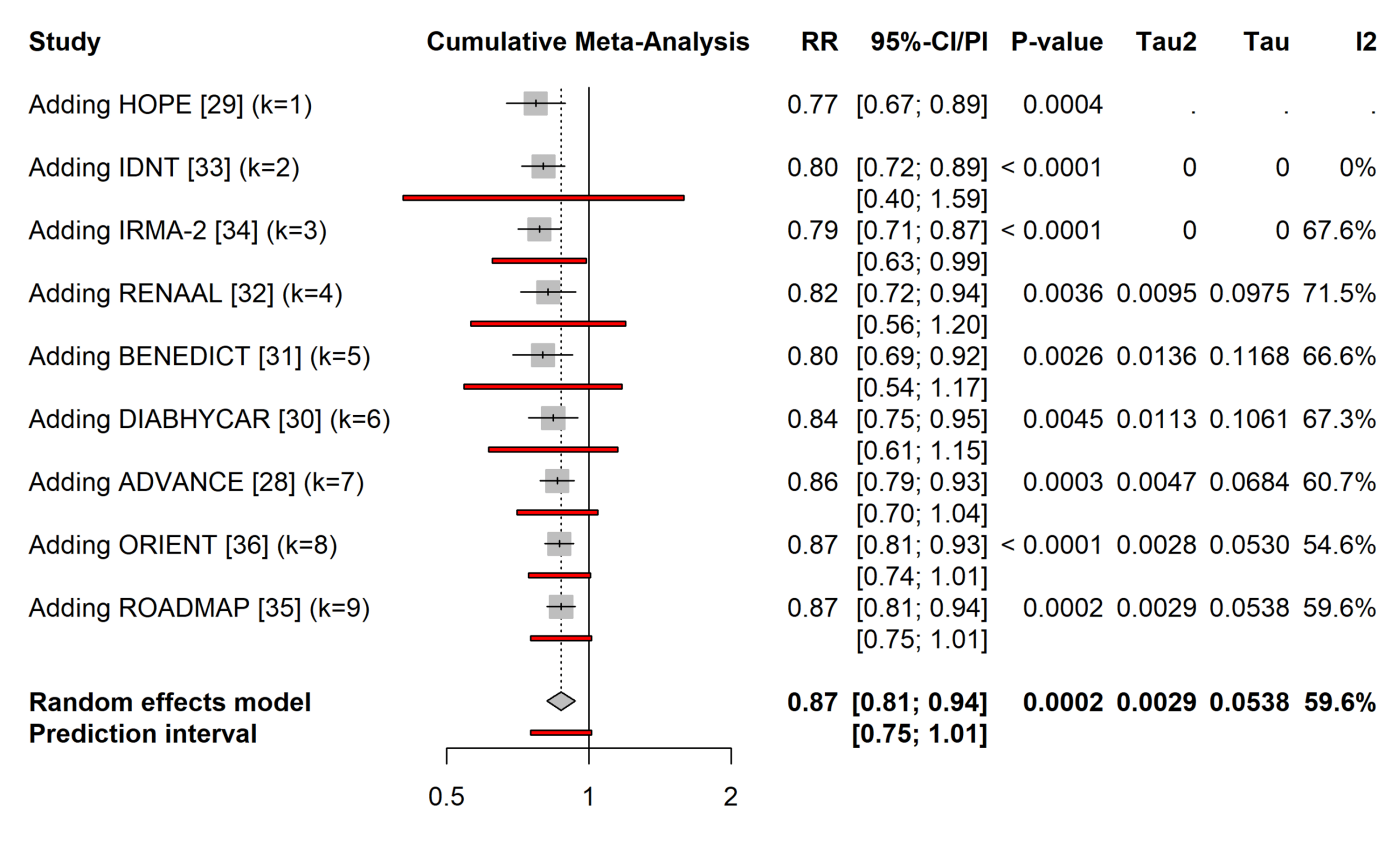
Cumulative meta-analysis. Forest plot showing the evolution of the summary RR over time as new studies were published (sorted by publication year). The term “Adding” on the y-axis indicates that the summary estimate on that line includes the specific study named plus all preceding studies listed above it, demonstrating how the evidence accumulated and stabilized over time. RR = risk ratio; CI = confidence interval

The cumulative meta-analysis (Figure 9) demonstrated that the beneficial effect of RAAS blockade became statistically significant and stable as early as 2000, following the publication of the HOPE trial [29]. Subsequent studies narrowed the CIs but did not materially change the point estimate.

**Figure 10:**
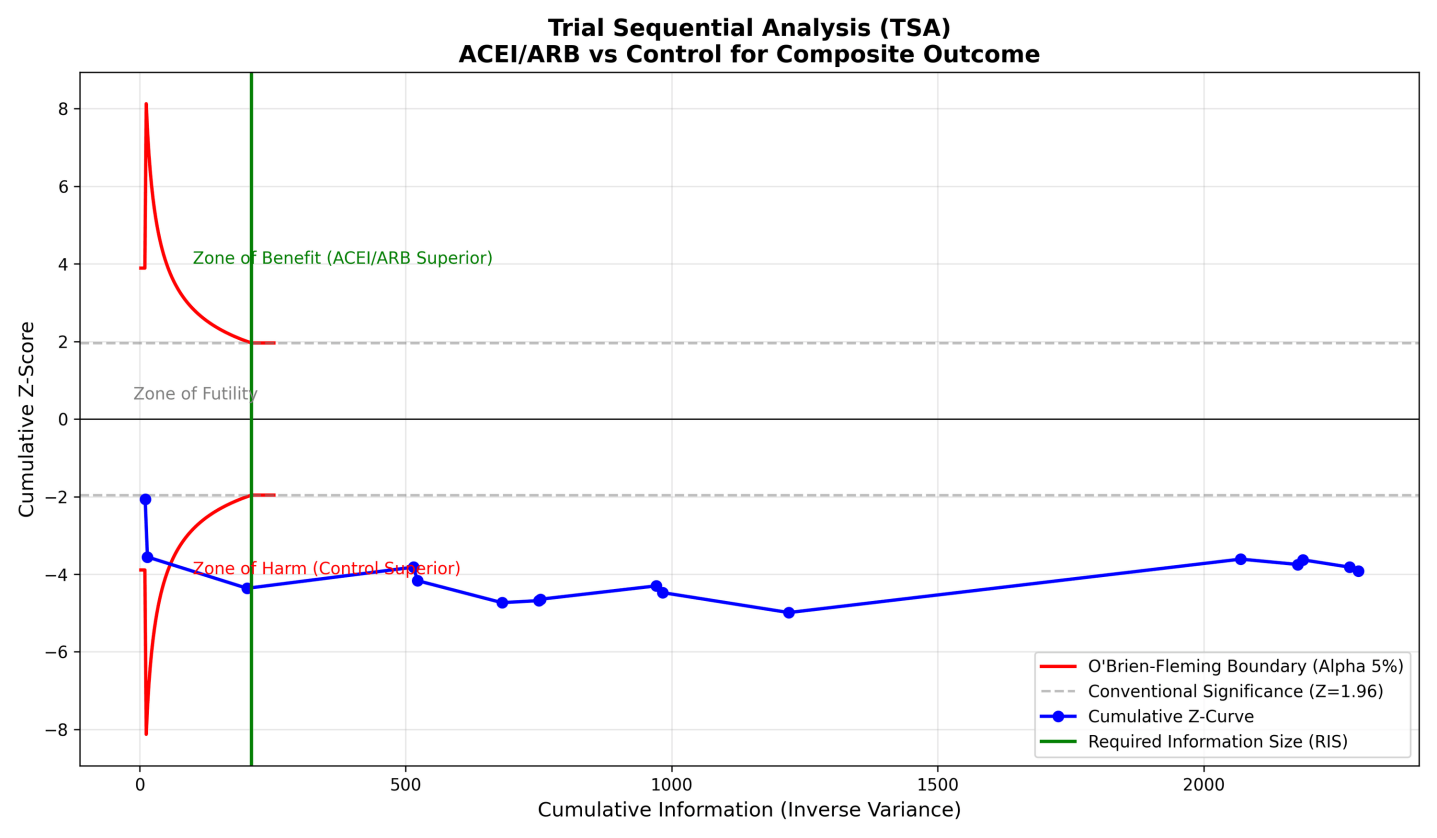
Trial sequential analysis (TSA). The cumulative Z-curve (blue) crosses the trial sequential monitoring boundary (red) and the required information size (green vertical line), indicating that the evidence for the benefit of renin-angiotensin-aldosterone system blockade is conclusive.

TSA was performed to determine whether the current evidence was sufficient. As shown in Figure 10, the cumulative Z-curve (blue line) crossed the conventional significance boundary (Z = 1.96) and the trial sequential monitoring boundary (red curve), entering the “Zone of Benefit.” Furthermore, the cumulative information size (x-axis) surpassed the required information size (green vertical line), confirming that the current body of evidence is conclusive and further placebo-controlled trials for this specific comparison are unlikely to change the result [23].

Discussion

This systematic review and meta-analysis provides a comprehensive synthesis of the evidence from 16 RCTs encompassing 47,406 patients, evaluating the relative efficacy of ACEIs and ARBs in cardiovascular and renal protection in T2DM. Utilizing stringent methodological standards, such as the RoB 2 assessment [12], Bayesian network meta-analysis [14], and TSA [23], the findings provide conclusive insights into the most effective therapy approach for this high-risk population.

Principal Findings

The primary analysis confirmed that RAAS blockade, by either ACEIs or ARBs, markedly reduced the risk of composite cardiovascular and renal events in comparison to placebo or active non-RAAS controls. ACEIs exhibited a 13% RRR (RR = 0.87; 95% CI = 0.81-0.94), while ARBs demonstrated a 10% reduction (RR = 0.90; 95% CI = 0.83-0.98). These findings align with current clinical guidelines advocating for RAAS inhibition as the cornerstone of therapy for diabetic nephropathy and hypertension [1,9].

A direct comparison of ACEIs and ARBs by NMA revealed no statistically significant difference in the primary composite endpoint (RR = 0.98; 95% CI = 0.88-1.09), suggesting therapeutic equivalence in preventing serious adverse events. While the SUCRA analysis ranked ACEIs higher than ARBs for reducing all-cause mortality (78% probability of being best vs. 65%), this finding must be interpreted with caution. The 95% credible intervals for the mortality difference were non-significant. Therefore, the SUCRA rankings reflect a probabilistic trend rather than robust clinical superiority. This observed trend may also be influenced by the era effect, as earlier ACEI trials (e.g., HOPE) often had lower rates of background statin use compared to later ARB trials, potentially amplifying the apparent relative benefit of the intervention in older studies.

Heterogeneity and Effect Modifiers

A critical finding from the meta-regression analysis was the significant influence of baseline SBP on the treatment efficacy (p = 0.04). A linear relationship was noted, suggesting that studies involving patients with elevated baseline SBP (e.g., IDNT, RENAAL, LIFE) exhibited greater RRRs compared to those with adequately managed baseline BP (e.g., ROADMAP). This baseline risk effect highlights the hemodynamic mechanism of renoprotection, wherein the reduction of intraglomerular pressure is particularly beneficial in patients with systemic hypertension [5,33]. In normotensive populations, the benefits of RAAS blocking may be diminished, advising against the indiscriminate application of these agents exclusively for preventive indications in the absence of overt hypertension or albuminuria [35].

Clinical Implications of Combination Therapy

The analysis of the VA NEPHRON-D and ONTARGET trials highlighted the significant safety concern associated with dual RAAS inhibition (ACEI + ARB). Although combination therapy potentially offers more comprehensive RAAS inhibition, our data confirm that it significantly elevates the risk of hyperkalemia (RR = 2.8) and acute kidney injury (RR = 1.7) without yielding additional survival or renal benefits relative to monotherapy [25,26]. These results support the current guidelines that discourage the routine use of dual blockade in T2DM patients.

Study strengths

The strength of this review lies in its rigorous methodology. The application of the RoB 2 tool guaranteed that our conclusions were based solely on high-quality evidence, with 81% of the studies rated as having a low risk of bias. In addition, the use of TSA confirmed that the cumulative Z-curve crossed the monitoring boundary for benefit, signifying that the existing evidence is conclusive and that additional placebo-controlled trials of RAAS blockade in this population are unnecessary and potentially unethical.

Limitations

The limitations of our study encompass the moderate heterogeneity observed in the ACEI mortality analysis (I² = 59.6%), indicative of the diverse patient populations (primary vs. secondary prevention) and varying follow-up durations (2.0 to 5.6 years) across the studies analyzed. Additionally, during our evaluation of renal outcomes, the definition of “nephropathy progression” differed between studies (e.g., doubling of serum creatinine vs. progression to macroalbuminuria), thus introducing inconsistency in the extent of renal improvement [34,36]. The analysis was restricted to aggregate study-level data; an individual patient data meta-analysis could provide more granular insights into subgroup effects, particularly in patients with normoalbuminuria.

Additionally, the vintage of the included trials (published largely between 1998 and 2013) introduces a potential bias related to evolving standards of care. Contemporary management of T2DM involves widespread use of high-intensity statins, sodium-glucose co-transporter 2 inhibitors, and glucagon-like peptide-1 receptor agonists, which were not standard in many of the included trials. This variation in background therapy across the decades constitutes a potential violation of the transitivity assumption in the network meta-analysis and limits the generalizability of ARR estimates to modern practice.

## Conclusions

This systematic review and meta-analysis establishes that RAAS inhibitors, specifically ACEIs and ARBs, are effective first-line interventions for reducing cardiovascular and renal morbidity in patients with T2DM. While both drug classes demonstrate therapeutic equivalence regarding the primary composite endpoint of cardiovascular death, myocardial infarction, stroke, and renal failure, the evidence suggests a subtle divergence in secondary outcomes, where ACEIs ranked higher in probability for mortality reduction, though statistical equivalence between the classes remains the primary finding. ARBs are associated with a better tolerability profile. Because TSA indicates the evidence base for RAAS blockade versus placebo is conclusive, further placebo-controlled trials of these agents are unnecessary and potentially unethical. Future research should focus on optimizing the combination of RAAS inhibitors with newer agents, such as SGLT2 inhibitors and non-steroidal mineralocorticoid receptor antagonists (e.g., finerenone), to mitigate residual cardiovascular and renal risk in this population.
